# Supplementary material for: A disorder-enhanced quasi-one-dimensional superconductor
Source: Nat Commun. 2016 Jul 22;7:12262. doi: 10.1038/ncomms12262 (PMC4961838; doi:10.1038/ncomms12262)
Supplement: Supplementary Information — Supplementary Figures 1-8, Supplementary Tables 1-2, Supplementary Notes 1-9 and Supplementary References [file ncomms12262-s1.pdf]

SUPPLEMENTARY FIGURES

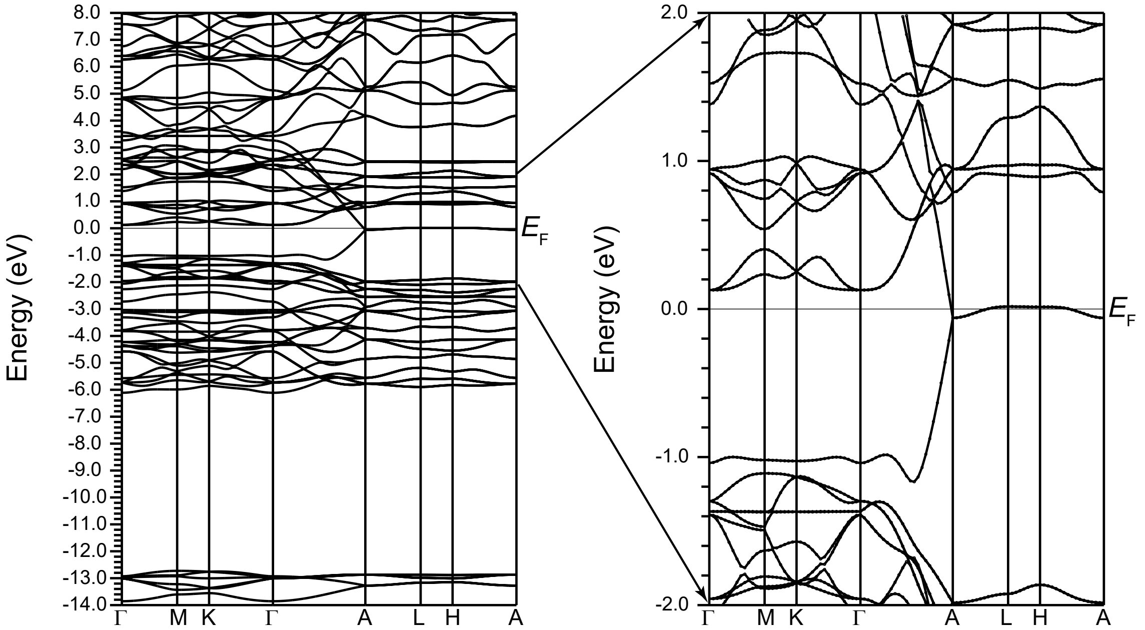

Supplementary Figure 1: **Electronic structure of $Na_2Mo_6Se_6$**

Density functional theory calculations of the band structure for $Na_2Mo_6Se_6$, obtained using the full-potential linear augmented-plane-wave method. The internal atomic co-ordinates were obtained from X-ray diffraction measurements; full details of the calculations may be found in Supplementary Note I and references [1, 2]. A zoom view of the region ± 2 eV around the Fermi level E_F highlights the single spin-degenerate Mo d_{xz} band crossing E_F. The quasi-one-dimensional nature of this band is immediately apparent: its dispersion is strong parallel to the chain axis (along the ΓA direction), but very weak perpendicular to the chains on the Brillouin zone boundary (AL-LH-HA).

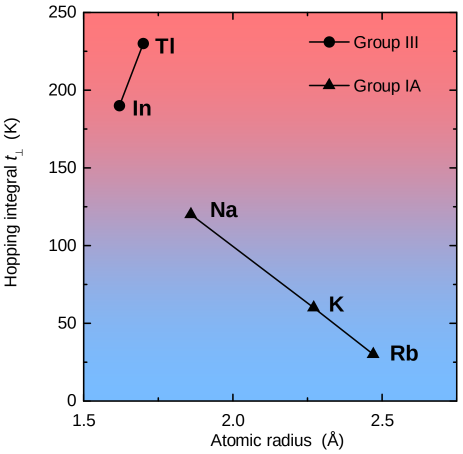

Supplementary Figure 2: **Evolution of the electronic anisotropy in $M_2Mo_6Se_6$**
The transverse hopping integrals $t_\perp$ are calculated from density functional theory (Supplementary Note I). Red and blue shading indicate superconducting and insulating ground states respectively.

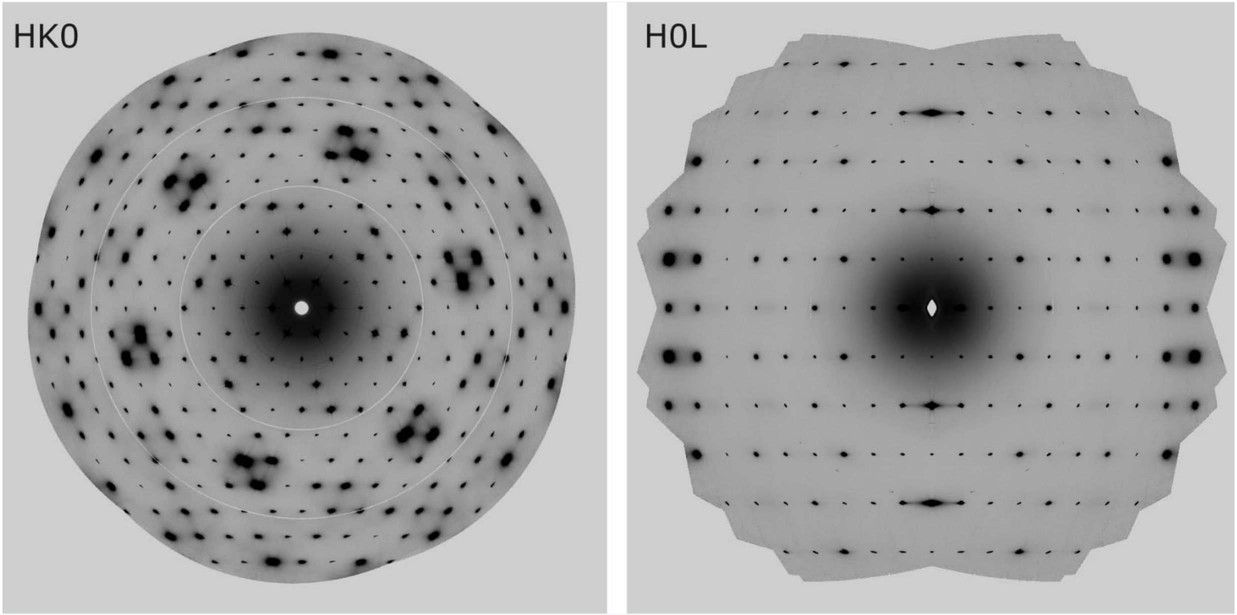

Supplementary Figure 3: **Diffuse X-ray scattering in $Na_{2-\delta}Mo_6Se_6$**
Reconstructed layer diffraction patterns from images acquired at 300 K in the $(hk0)$ (left) and $(h0l)$ (right) planes in a $Na_{2-\delta}Mo_6Se_6$ crystal with $\delta = 0.26$. The increased blurring in the Bragg spots for larger reciprocal lattice vectors is a signature of thermal diffuse scattering.

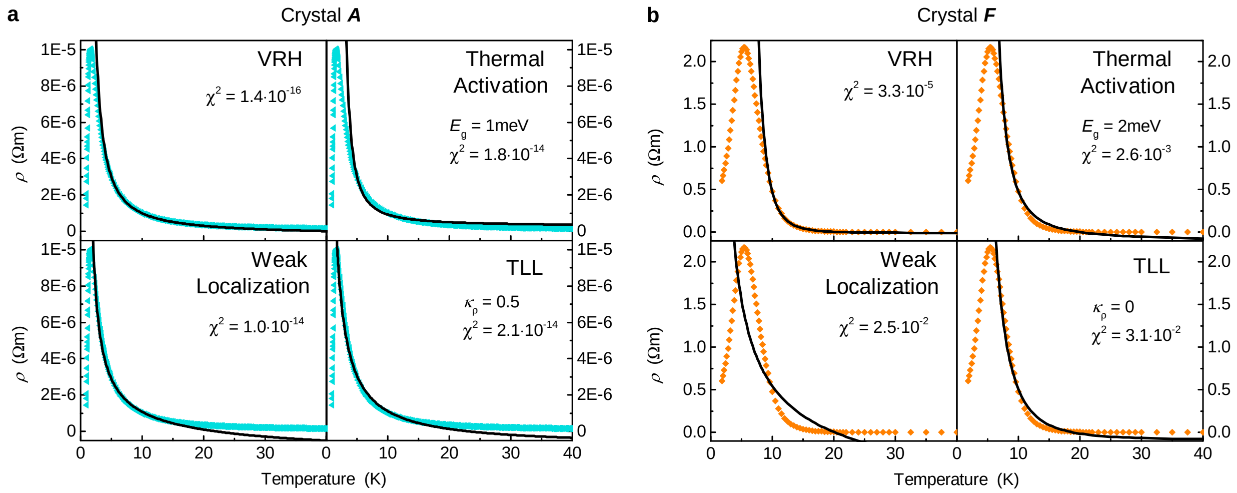

Supplementary Figure 4: **Mechanisms for divergent resistivity in $Na_{2-\delta}Mo_6Se_6$**
A selection of fits to the electrical resistivity $\rho(T)$ is shown for our least (**a**) and most (**b**) disordered crystals A and F. We model $\rho(T)$ using four distinct transport scenarios: variable range hopping (VRH, Supplementary equation 1); thermal activation across an energy gap (Arrhenius-like behaviour, Supplementary equation 2); weak localisation (Supplementary equation 3); repulsively-interacting Tomonaga-Luttinger liquid (TLL, Supplementary equation 4). The χ^2 values serve to quantify the goodness of fit. The experimental data and VRH fits are identical to those shown in Fig. 3a in the main text.

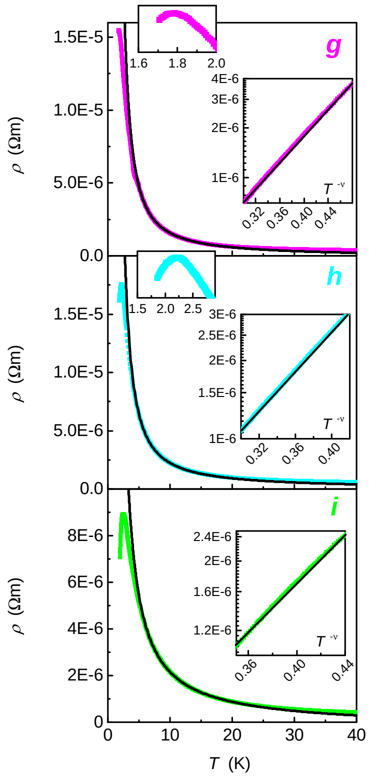

Supplementary Figure 5: **Additional electrical transport data from fragile crystals**
Electrical transport $\rho(T)$ in three crystals g, h, i which cracked during thermal cycling early in our measurement series. These data were used to obtain the three black data-points in Fig. 5c from the main text. The small insets for crystals g, h highlight the peak at $T_{\rm pk}$, while the linear behaviour of $\log \rho$ vs. $T^{-\nu}$ in the larger insets for all three crystals illustrates variable range hopping transport.

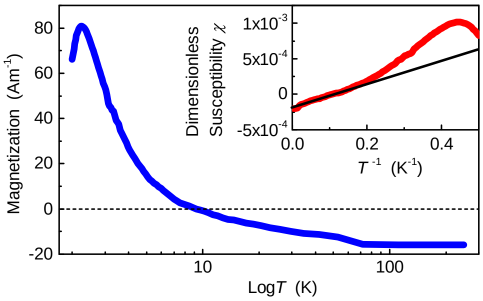

Supplementary Figure 6: **Emergent paramagnetism is an indicator for strong localisation**
Magnetisation at $T < T_{\rm min}$ for crystal E showing an emergent paramagnetism due to localised states. Inset: $\chi(T^{-1})$ at low temperature where a non-linear behaviour (i.e. a non-constant spin density) is clearly visible, thus excluding paramagnetic impurities from causing this effect. The downturn at lower temperature is due to the onset of superconductivity.

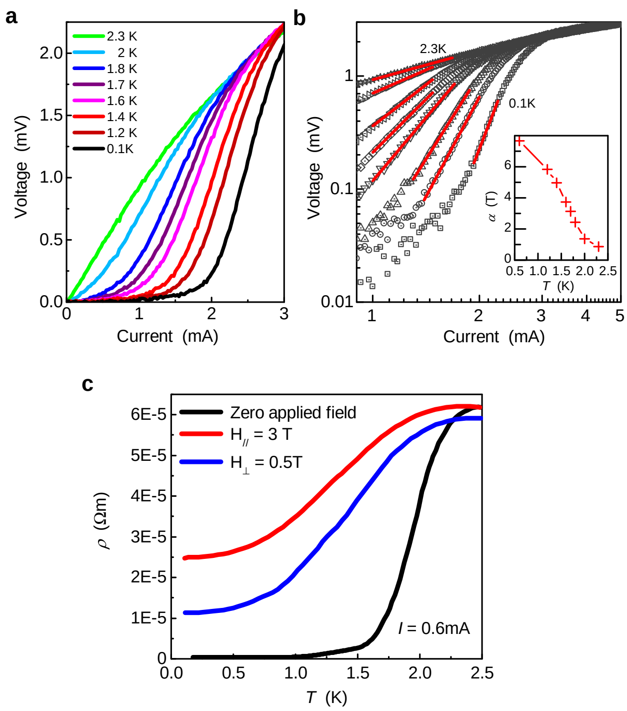

Supplementary Figure 7: **Establishment of bulk phase coherence via inter-chain coupling in quasi-one-dimensional $Na_{2-\delta}Mo_6Se_6$**

a Voltage-current $V(I)$ curves at a range of temperatures below $T_{\rm pk} = 2.7$ K in crystal C, which lies on the metallic side of the q1D mobility edge. **b** The same $V(I)$ data plotted on logarithmic axes. Red lines are power-law fits: $V \sim I^{\alpha}$. Inset: temperature evolution of the power-law exponent α. The condition $\alpha = 3$ commonly used to define the onset of phase coherence in 2D systems corresponds to the two-particle inter-chain hopping temperature $T_{\rm J} = 1.73$ K. **c** Anisotropic influence of magnetic fields on the superconducting transition in crystal C. We estimate the anisotropy by defining the upper critical field $H_{c2//,\perp}(T) \equiv T_c(H_{//,\perp})$ as the temperature at which the resistivity $\rho(T)$ falls to 80% of its value above the transition.

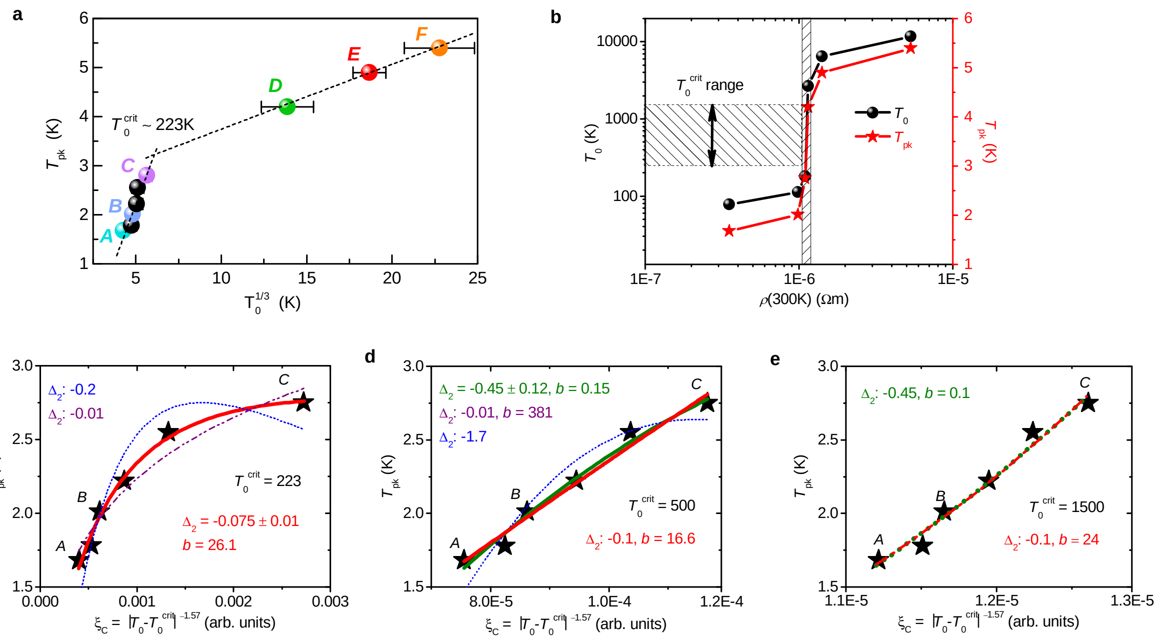

Supplementary Figure 8: **Multifractal pairing enhancement on the metallic side of the mobility edge**

a Superconducting onset temperature $T_{\rm pk}$ vs. $T_0^{1/3}$, obtained from variable range hopping (VRH) fitting. Data from crystals g, h, i are represented by black circles. The dashed lines are linear interpolations of the trends in $T_{\rm pk}$ for crystals above and below the mobility edge. Error bars correspond to the standard deviation in T_0 from our VRH fitting. **b** Evolution of T_0 and $T_{\rm pk}$ with the room temperature resistivity ρ(300K). Data are identical to those in Fig. 5b,c in the main text. The approximate range of critical disorder $T_0^{\rm crit}$ values in which the mobility edge may lie is indicated using hatched shading. **c** Variation of $T_{\rm pk}$ with the effective correlation length (Supplementary equation 18) for three values of $T_0^{\rm crit}$, together with fits using a multifractal enhancement model (Supplementary equation 19). Where quoted, errors are taken from unconstrained least-squares fitting routines. Δ_2 is the multifractal exponent describing the spatial correlation of the electron wavefunction amplitudes. $\Delta_2 : X$ implies that Δ_2 was manually fixed at X, leaving only a and b as unconstrained variables during fitting. For $T_0^{\rm crit} = 500$ K, the purple $\Delta_2 : -0.01$ curve is completely hidden behind the $\Delta_2 = -0.45$ and $\Delta_2 : -0.1$ fits.

SUPPLEMENTARY TABLES

Supplementary Table I: **Variable Range Hopping (VRH) parameters and the onset of superconductivity in $Na_{2-\delta}Mo_6Se_6$**

d and T_0 are acquired using a least-squares fitting technique and their errors correspond to the standard deviations from these fits. The VRH exponent $\nu = (1+d)^{-1}$.

Crystal	d	T_0 (K)	Fit range (K)	$T_{\rm pk}$ (K)
A	1.3±0.1	78±10	4.5-17.4	1.68±0.1
B	1.4±0.1	112±20	4.6-17.2	2.01±0.1
C	1.5±0.1	180±30	6.0-28.0	2.75±0.1
D	1.2±0.1	2670±990	7.0-27.0	4.2±0.1
E	1.3±0.1	6500±1050	8.4-23.6	4.9±0.1
F	1.6±0.1	11800±3500	9.8-26.4	5.4±0.1
g	1.3±0.1	108±22	4.8-17.5	1.78±0.1
h	1.4±0.1	129±31	4.6-17.6	2.22±0.1
i	1.7±0.1	134±36	5.0-20.0	2.55±0.1

Supplementary Table II: **Phase slip fit parameters for crystals** A**-**F

Fits are shown in Fig. 4a-i in the main text; further details of the combined thermal and quantum phase slip model may be found in Supplementary Note V.

Crystal	$A_{\rm Q}$	$B_{\rm Q}$	$Lm/n\xi$
A	1.99	19400	0.0145
B	1.81	2330	0.0120
C	0.607	44.4	0.0053
D	0.176	0.379	435
E	0.118	0.385	603
F	0.150	0.337	294

SUPPLEMENTARY NOTES

Supplementary Note I. Electronic Structure

Ab initio density functional theory (DFT) calculations have been reported for several members of the $M_2Mo_6Se_6$ family [1] and we have recently published a detailed summary of our calculations for $Na_2Mo_6Se_6$ [2]. In the present manuscript, we provide a brief synopsis of the electronic structural details for $Na_{2-\delta}Mo_6Se_6$ which are helpful to understand the transport measurements in the main text. For further information, we direct interested readers to our existing work [1, 2].

The main features of the band structure are very similar in all compounds of the $M_2Mo_6Se_6$ family. We plot the band structure of $Na_2Mo_6Se_6$ in Supplementary Fig. 1: a single spin-degenerate band of Mo d_{xz} character crosses the Fermi level E_F at the Brillouin zone boundary. The conduction band is highly dispersive in the direction parallel to the $(Mo_6Se_6)_\infty$ chains, yet weakly dispersive perpendicular to them: herein lies the origin of the extreme electronic anisotropy in $Na_2Mo_6Se_6$. We quantify this anisotropy using the ratio of the hopping integrals parallel ($t_{//}$) and perpendicular ($t_\perp$) to the chain axis: $t_{//}/t_\perp \sim 120$. This figure is considerably higher than in other well-known quasi-one-dimensional (q1D) materials: for example, $t_{//}/t_\perp \sim 45$ in $Li_{0.9}Mo_6O_{17}$ [3] and $t_{//}/t_\perp \sim 10$ in $(TMTSF)_2ClO_4$ [4].

Returning to the $M_2Mo_6Se_6$ family, we note that the key parameter differentiating its members is the inter-chain hopping $t_\perp$. In Supplementary Fig. 2, we plot the evolution of $t_\perp$ as the atomic radius of the intercalant M ion increases. As $t_\perp$ is reduced and the materials become more one-dimensional, the ground state changes. $Tl_2Mo_6Se_6$ and $In_2Mo_6Se_6$ are superconductors [1, 5–7], while $K_2Mo_6Se_6$ and $Rb_2Mo_6Se_6$ are insulating at low temperature [1, 8, 9].

Before the present series of measurements revealing a superconducting instability, the ground state in $Na_{2-\delta}Mo_6Se_6$ crystals had remained uncertain. Within the conventional Bardeen-Cooper-Schrieffer theory of superconductivity, the critical temperature T_c is proportional to the density of states (DoS) at E_F. One naturally wonders whether removing Na ions will influence the band structure and hence the DoS; it is therefore important to consider any change in the DoS as a potential cause of the variation in the superconducting onset temperature T_{pk} in our crystals. We find that in a rigid-band picture, reducing the Na content has no effect on the DoS, for two reasons. Firstly, the perpendicular dispersion of the conduction band is tiny compared with its parallel dispersion; secondly, the parallel dispersion is highly linear below E_F. The DoS is therefore a low yet constant $1/W \equiv 0.135$ states eV^{-1} $spin^{-1}$ per $NaMo_3Se_3$ unit, where $W = 2\pi t_{//}$ is the

conduction bandwidth (Fig. 1d in the main text). This relation holds from approximately $Na_{1.5}$ to $Na_{2.1}$, far beyond our experimentally-observed Na concentrations. Our observed enhancement of $T_{\rm pk}$ therefore cannot be explained by a change in the DoS due to varying Na stoichiometries.

Supplementary Note II. X-Ray diffraction measurements

X-ray diffraction experiments were performed at the Swiss-Norwegian Beamlines (SNBL) of the European Synchrotron Radiation Facility (Grenoble, France) at the end station BM01A, using a PILATUS2M pixel area detector [10]. Data were preprocessed by the SNBL Tool Box [11], followed by the CrysAlis Pro [12] software package. The crystal structure was solved with SHELXS and subsequently refined with SHELX [13]. Data were acquired at 293 K and 20 K: for both temperatures, we obtain a hexagonal lattice with space group $P6_3/m$, in agreement with previous reports [14, 15]. At 293 K, the lattice parameters are $a = 8.65$Å, $c = 4.49$Å (with a minimum inter-chain Mo-Mo separation of 6.4 Å), falling to $a = 8.61$Å, $c = 4.48$Å at 20 K.

We stress that we find no evidence for the crystal structure at 20 K deviating from that at 293 K. This rules out any Peierls-type (i.e. a dimerisation of the $(Mo_6Se_6)_\infty$ chains) or other structural distortion from occurring.

However, our XRD experiments do indicate a Na deficiency in our superconducting $Na_{2-\delta}Mo_6Se_6$ crystals: we measure $\delta = 0.2 \pm 0.036$, $\delta = 0.22 \pm 0.030$ and $\delta = 0.26 \pm 0.08$ in three randomly-chosen crystals. Since Na vacancies are expected to be the principle contributors to the disorder in our crystals, it is important to determine their spatial distribution: any correlation or ordering in the vacancy positions is incompatible with the random disorder responsible for localisation. A study of the diffuse X-ray scattering is therefore essential.

Diffuse X-Ray Scattering

Supplementary Fig. 3 displays reconstructed diffraction planes centred on $(0, 0, 0)$ for a $Na_{2-\delta}Mo_6Se_6$ crystal with 13% Na deficiency. The key feature in these images is a thermal diffuse scattering (TDS), i.e. a blurring in the Bragg spots which increases with the reciprocal lattice vector $\mathbf{Q}$. This is due to the TDS intensity scaling with $\mathbf{Q}\langle u\rangle$, where u is the average atomic displacement due to thermal lattice vibrations. The TDS intensity is controlled by structural factors and hence peaks near stronger Bragg reflections. Heating the crystal also increases the TDS intensity, as expected.

Let us now consider the contribution of Na vacancies to the diffraction patterns. Broadly, we can distinguish four possible scenarios:

1. Na vacancies exhibit long range order, which should be manifested as a set of new Bragg

reflections.

2. Na vacancies form clusters. This should result in an elastic distortion of the otherwise ideal (defect-free) matrix of elastic moduli. In a single crystal diffraction experiment this is seen as a temperature-independent Huang scattering (specific clouds of diffuse scattering near Bragg nodes, whose shape is set by the aforementioned matrix).

3. Long-range order is absent, but Na vacancies exhibit order at short lengthscales. This will lead to correlations such as chess-boards, planes, stripes, lines etc., but the correlation radius is rather short, i.e. only a few unit cells. In this case we should see structured diffuse scattering, such as periodic broad maxima, diffuse rods, pancakes, planes etc. in the diffraction images.

4. The disorder is entirely random. In this case, the diffuse scattering will give a monotonic background contribution which scales as the square of the Na form-factor and is virtually indistinguishable from the TDS in the rest of the crystal.

The X-ray scattering patterns which we acquire (Supplementary Fig. 3) are dominated by TDS and do not display any new Bragg reflections, Huang scattering or anomalous structures. This observation is consistent with case 4 above: the random disorder scenario. The apparent lack of any spatial correlations in the Na vacancy distribution is likely to originate from the elevated crystal growth temperature, together with a high Na ion mobility due to their small size and low mass. We therefore conclude that Na vacancies generate a random disorder potential within $Na_{2-\delta}Mo_6Se_6$ crystals and hence directly contribute to localisation.

Our X-ray data also permit us to assess the possibility of any In contamination in our crystals resulting from the Na/In ion exchange reaction during synthesis. The presence of any $In_2Mo_6Se_6$ or In-rich $(Na,In)_2Mo_6Se_6$ filamentary intergrowths can immediately be ruled out, since these would generate Huang scattering and disk-like Bragg reflections. No such features are observed in our scattering patterns, despite the extremely high intensity of the synchrotron X-ray source. We furthermore note that any In intermixing with Na would lead to an excess electron density at the Na crystallographic site, since In has a much larger atomic number than Na. Our results display the opposite behaviour: a strongly reduced electron density at the Na site corresponding to a Na deficiency. We therefore find no evidence for In contamination in our crystals using X-ray techniques.

Interestingly, the only experimental study of $Na_2Mo_6Se_6$ in the literature [16] reports superconductivity with a maximum T_c of 2.3 K to be induced by a small pressure ($\sim$ 50 kbar) in powder samples, although no raw data are provided to support this claim and measurements were not performed below 1.4 K. Powder samples are less likely than single crystals to exhibit Na deficiencies, since they are synthesised at lower temperatures. This early work is therefore consistent with our observed enhancement of superconductivity ($T_{pk} = 1.68 \rightarrow 5.4$ K) by disorder in Na-deficient single crystals.

Within this picture, an increase in the Na deficiency (i.e. more vacancies) constitutes an increase in the disorder. In principle, we could therefore tune $Na_{2-\delta}Mo_6Se_6$ across the critical disorder (indicated by the step in the variable range hopping temperature T_0 and T_{pk} at $\rho(300K) = 10^{-6}$ Ωm shown in Fig. 5b of the main text) by modulating the Na content. Since each Na atom donates 1 electron to the conduction band, removing Na from the crystals both reduces the Fermi energy and increases the disorder. The sharp jump in T_0 and T_{pk} between crystals C and D suggests that only a small change in the Na concentration may be required to shift the Fermi level across the critical disorder, i.e. the q1D mobility edge.

Unfortunately, even if we were able to accurately control the Na content during synthesis, the situation is unlikely to be quite so simple in real crystals. Intra-chain defects (e.g. impurity atoms or vacancies in the $(Mo_6Se_6)_\infty$ chains) will also efficiently localise the Mo d_{xz} conduction band electrons. Although our structural refinements indicate that the chains are highly ordered with 100% occupancies at the Mo and Se sites, we cannot exclude the possible influence of such defects in our crystals. This does not in any way affect the principal conclusion of our work: the positive correlation between superconductivity and disorder. Instead, we merely wish to highlight the possibility that changing δ alone may not be sufficient to continuously tune $Na_{2-\delta}Mo_6Se_6$ across the metal-insulator threshold.

Supplementary Note III. Modelling the divergent low-temperature resistivity in $Na_{2-\delta}Mo_6Se_6$

It is important to consider all possible causes for the insulating tendency (i.e. the divergent resistivity below $T_{\min}$) which we observe in $Na_{2-\delta}Mo_6Se_6$. A wide array of mechanisms can provoke a metal-insulator transition (see ref. [17] for a review), especially in the case of 1D crystal symmetry. We differentiate between these scenarios by quantitatively comparing their compatibility with our electrical transport data. Our four candidate models are introduced below:

1. Variable Range Hopping (VRH)

In a strongly disordered electron system, charge transport occurs by hopping between nearby localised states [18]. This results in the well-known VRH equation:

$$\rho(T) = \rho_0 \exp[(T_0/T)^{\frac{1}{1+d}}] \tag{1}$$

where T_0 is the effective localisation temperature, which describes both the hopping length and activation energy, and d is the dimensionality of the system. Caution is required when interpreting data-sets from low-dimensional materials, since $d = 1$ also describes the Efros-Shklovskii VRH for a system of arbitrary dimensionality in which Coulomb repulsion opens a soft (quadratic) gap [19] in the charge excitation spectrum at the Fermi level. In this case, a positive magnetoresistance would be expected.

2. Activated behaviour

Any phase transition which opens a hard gap in the density of states at the Fermi energy (i.e. an absence of any states over an energy range $E_{\rm g}$) will exhibit thermally-activated transport following the Arrhenius equation:

$$\rho \propto {\rm e}^{E_{\rm g}/k_B T} \tag{2}$$

Any density wave (DW) which gaps the entire Fermi surface will fall into this category. We note that a partial DW gapping a small segment of the Fermi surface is unlikely in $Na_{2-\delta}Mo_6Se_6$ due to the highly-nested, planar Fermi sheets; such a transition would also create a discrete jump in $\rho(T)$ rather than the continuous exponential divergence which we observe. Opening a gap at the Fermi level would furthermore lead to a gap developing in the frequency-dependent ac conductivity spectrum $\sigma(\omega)$: as can be seen in Fig. 3b from the main text, no such gap is present.

3. Weak localisation

We also consider the low disorder limit, in which weak localisation replaces strong (Anderson) localisation. Weak localisation is a disorder-induced quantum interference phenomenon which enhances backscattering for delocalised electrons, creating a logarithmically-divergent resistivity:

$$\rho = \rho_0 + \frac{\rho_1}{ln(T)} \tag{3}$$

where $\rho_{0,1}$ are constants. It should be noted that the weak negative magnetoresistance expected in the presence of weak localisation is suppressed in quasi-1D materials due to the open Fermi surface [20]. In contrast, negative magnetoresistance in the case of strong localisation remains unaffected by the material dimensionality [21] and has previously been observed [22] in q1D nanowires.

4. Tomonaga-Luttinger Liquid (TLL) with strong Coulomb repulsion

We finally consider a TLL with strongly repulsive electron-electron (e^--e^-) interactions, i.e. a Luttinger parameter $K_\rho \ll 1$. In this case, a power-law suppression in the density of states $N(E) \propto E^\alpha$ creates a pseudogap at the Fermi level and

$$\rho(T) \propto T^\alpha \tag{4}$$

with $\alpha = 4n^2K_\rho - 3 < 0$, where $n = 1$ for half-filling. Note that our data (Figs. 2,3 in the main text) indicate that the dominant e^--e^- interactions are attractive in $Na_{2-\delta}Mo_6Se_6$; furthermore, any TLL will become unstable to dimensional crossover below a renormalised temperature $T_x \leq t_\perp \equiv 120$ K. It is difficult to envisage any physical mechanism capable of both switching on repulsive interactions as the temperature falls and simultaneously preserving one-dimensional (TLL) behaviour far below T_x. Nevertheless, we attempt the fit for completeness, if only to verify whether $\rho(T)$ follows a power-law.

Our $\rho(T)$ data for the least (A) and most (F) disordered crystals are shown in Supplementary Fig. 4, together with VRH, Arrhenius, weak localisation and TLL fits. We have left all proportionality constants, exponents and scaling parameters (e.g. T_0, d, E_g, K_ρ, etc.) as completely free variables during our least-squares fitting procedure. For each model, we quantify the goodness of fit to our data using a standard Pearson χ^2 test: the χ^2 value resulting from our VRH fits is at least two orders of magnitude lower than its closest rival. We attribute the slight deviations between data and VRH fits at low temperature to a paraconductivity just above T_{pk} [23, 24]. Data from crystals B-E give similar results, with the Arrhenius fits deteriorating still further as the disorder

is reduced. We may draw two conclusions from these fits: electrons in $Na_{2-\delta}Mo_6Se_6$ are strongly localised and exhibit VRH transport, but no gap-forming instability develops as the temperature falls.

For reference, we include the key parameters and fitting ranges used in our VRH analysis (from Fig. 3a in the main text) in Supplementary Table I. $d \approx 1.5$ for all crystals (as expected from theoretical studies of coupled q1D conductors [25]) and exhibits no correlation with T_0, implying that the disorder has little or no effect on the dimensionality of $Na_{2-\delta}Mo_6Se_6$. Together, our observed negative magnetoresistance (Fig. 3d,e in the main text) and dimensionality $d > 1$ rule out Efros-Shklovskii Coulomb gap formation, indicating that the Coulomb repulsion is weak. We note that fixing d before fitting our $\rho(T)$ data would presuppose both the dimensionality and the strength of the Coulomb repulsion in $Na_{2-\delta}Mo_6Se_6$. It is therefore important to leave d as a free parameter: this is particularly relevant in localised systems which may exhibit fractal rather than integer dimensionality [26].

Finally, we note that our standard experimental $\rho(T)$ acquisition procedure necessitated rapidly cooling the crystals by inserting them into a cold cryostat, then gradually warming them to room temperature while collecting data. Several particularly small and fragile crystals (labelled g, h, i) did not survive thermal expansion during warming and cracked in the 50-200 K range. Nevertheless, their $\rho(T)$ curves at low temperature enabled us to identify T_{pk} and perform VRH fitting to extract T_0 (Supplementary Fig. 5). These parameters are listed in Supplementary Table I and correspond to the black data-points in Fig. 5c of the main text (as well as Supplementary Fig. 8). No deviation was observed from the trends shown by the larger surviving crystals.

Supplementary Note IV. Bulk magnetisation data: emergent paramagnetism coincides with localisation

In the metallic phase, $M_2Mo_6Se_6$ are weakly diamagnetic, with the susceptibility $\chi(T)$ roughly temperature-invariant [8, 27]. Measuring the magnetization of $Na_{2-\delta}Mo_6Se_6$ in a Quantum Design MPMS XL SQUID magnetometer, we observe a weak emergent paramagnetism developing below $T_{\rm min}$ after careful background subtraction (Supplementary Fig. 6). This cannot be attributed to paramagnetic impurities in the crystal for two reasons. Firstly, a plot of $\chi(T)$ vs. T^{-1} is not linear at low temperature (Supplementary Fig. 6 inset), with $d^2\chi/d(T^{-1})^2 > 0$ (the peak and subsequent downturn in $\chi(T^{-1})$ is due to the onset of superconductivity). This implies that the paramagnetic spin density increases at low temperature, in contrast with the expected constant density and linear $\chi(T^{-1})$ from impurities. Secondly, paramagnetism only develops below $\sim T_{\rm min}$ and $\chi(T)$ remains constant at higher temperatures: paramagnetic impurities would be visible as $\chi \propto 1/T$, independently of temperature.

Instead, we may interpret this emergent paramagnetism as a signature of localisation. The half-filled band in $Na_2Mo_6Se_6$ is principally ($> 90\%$) of Mo d_{xz} character, leading to a low unpaired electron density. In the metallic phase ($T > T_{\rm min}$), the resultant Pauli paramagnetic contribution is far smaller than the Larmor and Landau diamagnetism, leading to the weak diamagnetism common to all members of the $M_2Mo_6Se_6$ family. Below $T_{\rm min}$, electrons are progressively localised: the Landau diamagnetism vanishes and there is a gradual crossover from the small, free electron-like Pauli contribution to a large $1/T$ Curie paramagnetism from localised electrons. We note that similar behaviour has previously been observed in other strongly localised materials [28].

It is also important to verify the presence of any spin density wave (SDW) at low temperature, since SDWs are a common instability in q1D materials. SDW formation implies antiferromagnetic ordering along the $(Mo_6Se_6)_\infty$ chains, which should create a peak in $\chi(T)$ (and a gap in the spin excitation spectrum). The only peak visible in $\chi(T)$ corresponds to the onset of superconductivity and we hence can rule out any magnetic ordering in $Na_{2-\delta}Mo_6Se_6$.

Supplementary Note V. Phase slips in one-dimensional superconductors

An ideal 1D superconductor - such as a nanowire with diameter $d \ll \xi$, where ξ is the Ginzburg-Landau (GL) coherence length - does not reach a phase-coherent state due to fluctuations. At $T < T_{\mathrm{pk}}$, the superconducting order parameter may fluctuate to zero at some point along the wire, allowing the phase to slip by 2π, creating a resistive state. We distinguish two separate origins for these phase slips: thermal activation and quantum fluctuations.

The theory describing thermally activated phase slips (TAPS) was developed by Langer, Ambegaokar, McCumber and Halperin (LAMH) [29, 30]. Here, phase slips are thermally activated over an energy barrier ΔF, proportional to $\xi(T) = \xi(0)(1 - T/T_{\mathrm{pk}})^{-1/2}$ and the length of the nanowire L. The time scale of the fluctuations is fixed using a prefactor Ω, related to the attempt frequency of random excursions in the superconducting order parameter. The LAMH contribution to the total resistance can be expressed as follows:

$$R_{\mathrm{LAMH}}(T) = \frac{\pi\hbar^2\Omega}{2e^2 k_{\mathrm{B}}T}\exp\left(\frac{-\Delta F}{k_{\mathrm{B}}T}\right) \tag{5}$$

where the attempt frequency is given by:

$$\Omega = \frac{L}{\xi}\left(\frac{\Delta F}{k_{\mathrm{B}}T}\right)^{1/2}\frac{1}{\tau_{\mathrm{GL}}} \tag{6}$$

and $\tau_{\mathrm{GL}} = [\pi\hbar/8k_{\mathrm{B}}(T_{\mathrm{pk}} - T)]$ is the GL relaxation time. Following a development of the energy barrier by Lau *et al.* [31], we can write ΔF as

$$\Delta F(T) = Ck_{\mathrm{B}}T_{\mathrm{pk}}\left(1 - \frac{T}{T_{\mathrm{pk}}}\right)^{3/2} \tag{7}$$

where C is a dimensionless parameter relating the energy barrier for phase slips F to the thermal energy near T_{pk}:

$$C \approx 0.83\left(\frac{L}{\xi(0)}\right)\left(\frac{R_{\mathrm{q}}}{R_{\mathrm{F}}}\right) \tag{8}$$

Here, $R_{\mathrm{q}} = h/4e^2 = 6.45k\Omega$ is the resistance quantum for Cooper pairs and R_{F} the normal state resistance of the *entire* nanowire [31, 32].

We have recently generalised the LAMH model to describe macroscopic q1D crystals as well as single nanowires [2]. The crux of the argument is that we model the crystal as a $m \times n$ array of identical parallel nanowires, each of length L. In Supplementary equation 8, this leads to the replacement of R_{F} by the total crystal resistance R_{N} as well as a geometric renormalisation of L to Lm/n, where Lm is the experimental voltage contact separation on a crystal and n is the

typical number of 1D filaments within the crystal cross-section. LAMH theory can therefore remain applicable beyond the single nanowire limit.

Within the present data-set, thermal phase slips are expected to be the principal contributors to the resistivity in *less-disordered* crystals close to $T_{\rm pk}$. However, the influence of thermal phase slips tends to zero as the temperature falls. In contrast, quantum phase slips (QPS) arise due to quantum rather than thermal fluctuations in the order parameter and can therefore persist even as $T \to 0$. The QPS contribution to the resistivity in a 1D superconductor becomes relevant when $k_{\rm B}T < \Delta(T)$, where $\Delta(T)$ is the superconducting gap and $\Delta(T=0) \equiv \Delta_0$. For weakly-coupled superconductors ($\Delta_0 = 1.76k_{\rm B}T_{\rm pk}$), this corresponds to the temperature range $T < 0.86T_{\rm pk}$.

In crystals *D-F* with super-critical disorder, we anticipate that QPS will play an increasingly important role in the resistive transitions, for two reasons. Firstly, the probability for QPS formation is increased in the presence of strong disorder [32]. Secondly, the pairing enhancement which we infer from our magnetoresistance data (Fig. 5d-g in the main text) corresponds to a rise in Δ_0 beyond the weak-coupling limit $1.76k_{\rm B}T_{\rm pk}$. Upon cooling below $T_{\rm pk}$, the experimental condition for QPS detection $\Delta(T) > k_{\rm B}T$ is achieved at a higher absolute temperature *and* at a larger fraction of $T_{\rm pk}$ than for the less-disordered (weak-coupling) case. QPS may therefore influence $\rho(T)$ over a broader temperature range (i.e. closer to $T_{\rm pk}$) in strongly-disordered crystals.

The QPS contribution to the resistivity of a superconducting nanowire may be modelled using the relation [33]:

$$R_{\rm QPS} = A_{\rm Q}B_{\rm Q}\frac{R_{\rm q}^2}{R_{\rm F}}\frac{L^2}{\xi_0^2}\exp[-A_{\rm Q}\frac{R_{\rm Q}}{R_{\rm F}}\frac{L}{\xi(T)}] \tag{9}$$

where $A_{\rm Q}$ and $B_{\rm Q}$ are constants. In a similar manner to the TAPS contribution, we treat our crystals as macroscopic arrays of nanowires and rewrite equation 9 in terms of Lm/n and the resistance of the entire crystal. (Note that this step eliminates any unphysical disappearance of the resistance for large L.) Finally, the total resistance is evaluated by summing these two phase slip terms and adding the quasiparticle contribution $R_{\rm N}$:

$$R = (R_{\rm N}^{-1} + (R_{\rm LAMH} + R_{\rm QPS})^{-1})^{-1} \tag{10}$$

We performed least-squares fits to our experimental $\rho(T)$ transitions for $T < T_{\rm pk}$ in crystals *A-F*, using Supplementary equations 5,9,10 with $A_{\rm Q}$, $B_{\rm Q}$ and $Lm/n\xi$ as free parameters (listed in Supplementary Table II). The resultant curves are shown in Fig. 4a-i in the main text. In q1D superconductors whose resistance is influenced by QPS, $A_{\rm Q}$ is expected to be of order unity, in agreement with our data. However, $A_{\rm Q}$ is larger in the less-disordered crystals $A - C$, implying

that the QPS rate falls [34]: $\rho(T)$ is therefore dominated by TAPS in the limit of weak disorder. B_{Q} is proportional to the effective length of the 1D filaments [34] and should hence fall as the disorder rises: this is also apparent from our fit parameters.

Conversely, $Lm/n\xi$ rises by a factor of 10^5 between crystals C and D. Two features contribute to this effect: firstly, ξ falls as the disorder increases (as occurs in any dirty superconductor). However, a reduction in ξ alone cannot explain such a large increase in $Lm/n\xi$. Instead, we principally attribute this effect to a spatial inhomogeneity developing in the pairing interaction upon crossing the q1D mobility edge [35–38].

This emergent inhomogeneity creates small islands of superconductivity within a localised sea, drastically reducing the number of parallel superconducting filaments n within a typical cross-section of the crystal. Electrical transport across such an inhomogeneous superconducting material is highly percolative, resulting in a further reduction to the effective n within our model (since only a small fraction of the filaments will actually contribute to transport). This reduction in $Lm/n\xi$ due to inhomogeneity provides further justification for the unusually small diamagnetic susceptibilities which we measure (Fig. 4g,h,j in the main text), corresponding to a superconducting volume fraction of $\sim 0.1\%$. Unfortunately, we cannot quantitatively compare this value of 0.1% with the rise in $Lm/n\xi$: all superconducting zones in the crystal will contribute to the measured volume fraction (compared with a fraction of all filaments participating in transport) and Josephson-coupled superconducting networks may screen internal non-superconducting regions.

In summary, TAPS dominate the resistivity immediately below T_{pk} in the weakly-disordered crystals $A - C$ and the contribution from QPS is almost negligible. However, the influence of QPS rises strongly after crossing the mobility edge to crystals D-F. Together with the emergent spatial inhomogeneity in the pairing interaction, these QPS substantially broaden the superconducting transitions on the insulating side of the mobility edge. In particular, we note that $d\rho/dT|_{2\mathrm{K}}$ falls as the disorder rises, as expected for an increasingly large QPS resistive component persisting to low temperature.

Supplementary Note VI. Establishment of phase coherence in $Na_{2-\delta}Mo_6Se_6$

It is important to consider the distinction between the onset of Cooper pairing and the establishment of bulk phase coherence in our crystals. Previously, it has been suggested that although the pairing energy may be increased by disorder, the phase stiffness invariably falls, thus *reducing* the phase coherence temperature and hence the true superconducting transition [35]. We will now demonstrate that this does not occur in $Na_{2-\delta}Mo_6Se_6$: disorder enhances both the pairing and phase coherence temperatures.

The relevance of phase fluctuations at a superconducting transition is dictated by the Ginzburg number G_i, where $G_\mathrm{i}T_\mathrm{pk}$ determines the temperature range around the pairing temperature T_pk which is dominated by phase fluctuations. Although our present data-set does not allow us to evaluate G_i in $Na_{2-\delta}Mo_6Se_6$, we may estimate the order of magnitude for G_i by comparison with previous results in $Tl_2Mo_6Se_6$ and $In_2Mo_6Se_6$. Using an anisotropic 3D model, $G_\mathrm{i}^\mathrm{3D} = 1.8\times10^{-5}$ in $Tl_2Mo_6Se_6$ [39], while a 1D model designed for individual nanowires [40] yields $G_\mathrm{i}^\mathrm{1D} = 0.36$ and 0.69 for $Tl_2Mo_6Se_6$ and $In_2Mo_6Se_6$ respectively [1]. In $Na_{2-\delta}Mo_6Se_6$, G_i^1D and G_i^3D should characterise the phase stiffness at temperatures above and below the two-particle inter-chain hopping temperature T_J respectively: at T_J, bulk 3D (inter-chain) phase coherence is established via Josephson coupling. The extremely small values for G_i^3D imply that phase fluctuations in $Na_{2-\delta}Mo_6Se_6$ are negligible below T_J. A rise in phase fluctuations due to increased disorder would therefore correspond to a reduction in T_J for more disordered crystals.

From our electronic structure calculations, we estimate $k_\mathrm{B}T_\mathrm{J} \equiv t_\perp^2/t_{//} \sim 1$ K ($t_{//}$ is the hopping integral parallel to the MoSe chains). However, disorder may renormalise this value to higher temperature, either by reducing the effective $t_{//}$ or by enhancing electron-electron interactions [4]. Experimentally, we can identify the onset of bulk coherence (and hence T_J) via two separate techniques. Firstly, we examine the voltage-current ($V(I)$) curves acquired in crystal C at temperatures below T_pk (Supplementary Fig. 7a). It is clear that dV/dI only tends towards zero (a signature of inter-chain phase coherence) at small currents for $T < 2$ K.

Plotting the same data on logarithmic axes reveals power-law behaviour $V \sim I^\alpha$ over a broad temperature range (Supplementary Fig. 7b). The power-law exponent α rises as the temperature falls, similar to the behaviour seen in Berezinskii-Kosterlitz-Thouless (BKT) transitions in 2D materials [41]. Similarities between the 2D BKT transition and the onset of phase coherence in q1D superconductors have been noted in numerous studies [42–45], which have especially highlighted the duality between T_BKT in 2D and T_J in q1D systems. In 2D materials, the BKT transition

takes place at $\alpha(T_{\rm BKT} = 3)$: following the same definition in $Na_{2-\delta}Mo_6Se_6$ yields $T_{\rm J} = 1.73$ K. Another feature reminiscent of a BKT-style transition is visible in $\rho(T)$ for crystals $A - C$, which exhibit small humps below temperatures $T_{\rm J} \sim 0.95$ K, 1.25 K and 1.7 K respectively due to the establishment of Josephson coupling between the MoSe chains (Fig. 4d-f in the main text). Within a BKT scenario, such humps are caused by current-induced vortex unbinding and finite size effects [46].

The key point here is that the phase coherence temperature $T_{\rm J} = 1.73$ K in crystal C is higher than the pairing temperature $T_{\rm pk} = 1.68$ K in the least-disordered crystal A, which only develops interchain phase coherence at much lower temperature $T_{\rm J} \sim 0.95$ K (Fig. 4d in the main text). This indicates that both pairing *and* phase coherence are enhanced by disorder, in accordance with previous predictions [47–50].

In 2D systems, the phase fluctuation-induced offset between the pairing temperature and $T_{\rm BKT}$ is usually small in the weak disorder limit (i.e. low dimensionless resistance relative to h/e^2) [51]. We estimate the dimensionless resistance at $T_{\rm pk}$ in crystals $A - C$ to be $\sim 10^{-4}h/e^2$, and of the order of h/e^2 in crystals $D - F$. Accordingly, the offset between $T_{\rm pk}$ and $T_{\rm J}$ is observed to increase with disorder, ranging from ~ 0.7 K in crystal A to ~ 1.4 K in crystal E. Here, it is important to note that in q1D superconductors, a combination of strong pairing and high anisotropy can create large offsets between $T_{\rm pk}$ and $T_{\rm J}$ even for zero disorder.

The anisotropy of the superconducting ground state is revealed by comparing the influence of magnetic fields applied perpendicular and parallel to the chains (Supplementary Fig. 7c). As expected for q1D materials, superconductivity is more resilient to parallel fields, due to the short inter-chain coherence length. Assuming the upper critical field $H_{\rm c2}(T)$ rises linearly at small fields, we may make a rough estimate of the anisotropy, obtaining $H_{\rm c2//}/H_{\rm c2\perp} \sim 6.0$. A complete analysis of the temperature, field and current dependence of the superconducting transition in weakly-disordered $Na_{2-\delta}Mo_6Se_6$ may be found in Ref. [2].

In more disordered crystals on the insulating side of the q1D mobility edge, the small hump below $T_{\rm J}$ in $\rho(T)$ is smeared out by a large QPS contribution. However, our data from Fig. 4g,h,j in the main text show that $T_{\rm J}$ has been enhanced to sufficiently high temperature ~ 3 K to be detectable as a Meissner effect using a commercial SQUID magnetometer (which cannot operate below 1.8 K). We emphasise that a Meissner effect in q1D materials can only develop in the presence of Josephson coupling, i.e. transverse phase coherence. This confirms the enhancement of the bulk phase coherence temperature as well as the pairing energy by disorder.

In summary, pairing and local intra-chain coherence develop at temperature $T_{\rm pk}$ in

$Na_{2-\delta}Mo_6Se_6$. We note that pairing fluctuations may also exist above $T_{\rm pk}$ and could be responsible for the paraconductivity which deviates $\rho(T)$ from our VRH fits for $T \lesssim 1.5T_{\rm pk}$ (Fig. 3a in the main text). Directly below $T_{\rm pk}$, phase fluctuations are dominant (as is evident from the prevalence of phase slips in $\rho(T)$ and the large 1D Ginzburg number $G_{\rm i}^{\rm 1D}$). However, as the temperature is reduced further, a 1D→3D superconducting dimensional crossover occurs and bulk phase coherence mediated by Josephson coupling emerges at $T_{\rm J} < T_{\rm pk}$. Our data indicate that $T_{\rm J}$ and $T_{\rm pk}$ *both* rise as the disorder increases.

Supplementary Note VII. Temperature dependence of the Pauli limit

The Pauli paramagnetic limit (also known as the Clogston-Chandrasekhar limit [52]) for singlet-paired superconductors is reached when the energy cost of maintaining 50% of the Cooper pair spins at the Fermi level antiparallel to the applied field becomes equal to the difference in free energies between the normal and superconducting phases, $\Delta\mathcal{F}_{\rm NS} \equiv \mathcal{F}_{\rm N} - \mathcal{F}_{\rm S}$. We may express $\Delta\mathcal{F}_{\rm NS}$ in terms of the thermodynamic critical field $H_{\rm c}(T)$:

$$\frac{H_{\rm c}^2(T)}{8\pi} = \Delta\mathcal{F}_{\rm NS} \tag{11}$$

and for $T = 0$, $\Delta\mathcal{F}_{\rm NS} = 1/2 N_{\rm P}\Delta_0^2$ where $N_{\rm P}$ is the superconducting pair density and $\Delta_0 \equiv 1.76 k_{\rm B}T_{\rm c}$ is the zero-temperature BCS gap. $H_{\rm c}(T)$ is well-approximated by a parabola:

$$H_{\rm c}(T) = H_{\rm c}(0)\left(1 - \left(\frac{T}{T_{\rm c}}\right)^2\right) \tag{12}$$

and we may therefore write:

$$\Delta\mathcal{F}_{\rm NS}(T) = \frac{N_{\rm P}\Delta_0^2}{2}\left(1 - \left(\frac{T}{T_{\rm c}}\right)^2\right)^2 \tag{13}$$

Now, the energy cost of a 50% antiparallel spin population $\mathcal{F}_{\rm AP} = \frac{1}{2}\chi_{\rm P}H^2$, where the total paramagnetic susceptibility $\chi_{\rm P} = 2\mu_{\rm B}^2 N_\sigma$ ($\mu_{\rm B}$ is the Bohr magneton, N_σ is the density of states at the Fermi level per spin and we assume a Landé g-factor of 2). At the Pauli limit $H{\equiv}H_{\rm P}$, $\Delta\mathcal{F}_{\rm NS} = \mathcal{F}_{\rm AP}$, i.e. the energy saved by forming $N_{\rm P}(T)$ pairs is balanced by the energy cost of maintaining N_σ spins antiparallel to H. Since $N_{\rm P}(T) = N_\sigma(T) = \frac{1}{2}N_{E_{\rm F}}\left(1 - \frac{T}{T_{\rm c}}\right)$, we obtain

$$H_{\rm P}(T) = \frac{\Delta_0}{\sqrt{2}\mu_{\rm B}}\left(1 - \left(\frac{T}{T_{\rm c}}\right)^2\right) \tag{14}$$

Setting our experimentally-determined $T_{\rm pk}{\equiv}T_{\rm c}$, we may hence calculate a temperature-dependent Pauli limit for the phase-fluctuating one-dimensional superconductivity present in $\rm Na_{2-\delta}Mo_6Se_6$, even though the maximum field which we are able to apply (14 T) is only sufficient to suppress the onset of superconductivity to 84% of $T_{\rm pk}$ in crystal F.

Supplementary Note VIII. Multifractal enhancement of superconductivity

In recent years it has been proposed that in the absence of long-range Coulomb repulsion, T_c may rise in strongly disordered superconductors due to the emergent multifractality in the electron wavefunctions at the Anderson transition [37, 47, 49, 53]. Given the similarity between this scenario and our results (which demonstrate an enhancement of superconductivity by disorder in a material exhibiting evidence for a screened Coulomb repulsion), we believe it is worth analysing our data from a multifractal perspective.

The principles underlying multifractal T_c enhancement may be summarised as follows: at the localisation threshold, an electron wavefunction must simultaneously be spatially confined, yet still extend throughout a material to enable transport. To resolve this apparent contradiction, multifractalisation of the wavefunction occurs [54]. Uniformly spatially-distributed Bloch waves are replaced by multifractalised electron eigenfunctions, which only occupy a fraction of the volume within their correlation radii [47]. This leads to a mosaic-like spatial distribution for each electronic wavefunction, composed of finite fractal elements. Local peaks in this distribution enhance electron-electron correlations: if a superconducting instability is present, the pairing energy Δ and T_c may rise [37, 47, 49, 53]. So far, quantitative predictions for multifractal enhancement have only been made in 2D and 3D superconductors [53] rather than the q1D geometry presented by $Na_{2-\delta}Mo_6Se_6$. However, dimensional crossover at low temperature implies that $Na_{2-\delta}Mo_6Se_6$ is a highly anisotropic electron liquid which nevertheless experiences electronic correlations in *three* dimensions: multifractal enhancement is therefore physically plausible. [As an aside, we note that multifractality has previously been confirmed to develop in Anderson-localised 1D metals [55]. It would be interesting to investigate whether pairing enhancement is indeed possible in the 1D limit, e.g. a single disordered superconducting nanowire.]

On the metallic side of a mobility edge, the correlation length for electron density fluctuations ξ_C diverges as we approach the critical disorder. The enhancement of T_c has been calculated as a function of ξ_C using renormalisation group techniques [53]:

$$T_c = A\xi_C^{-3}\exp(-B\xi_C^{\Delta_2}) \tag{15}$$

where A and B are constants. To examine whether the rising T_{pk} in $Na_{2-\delta}Mo_6Se_6$ is compatible with a multifractal enhancement scenario, we must attempt to fit Supplementary equation 15 to our data from crystals *A*-*C*,*g*-*i* on the metallic side of the mobility edge (Fig. 5c). Any successful fit must use a realistic value for the multifractal exponent Δ_2, which describes the spatial correlation

of the electron wavefunction amplitudes and hence varies with dimensionality.

An accurate determination of ξ_C in $Na_{2-\delta}Mo_6Se_6$ is challenging. For the Anderson metal-insulator transition in disordered films or bulk 3D crystals, ξ_C can in principle be estimated from the conductivity using:

$$\sigma = \frac{e^2}{\hbar \xi_C^{d-2}} \tag{16}$$

However, Supplementary equation 16 is only strictly valid at $T = 0$, where our crystals are superconducting. We would therefore have to use data above T_{pk} in all our metallic crystals, e.g. at $T \gtrsim 3$ K. In fact, measuring at even higher temperatures would be safer due to the likely presence of pairing fluctuations above T_{pk}: it is unclear whether Supplementary equation 16 still gives accurate values for ξ_C at ~ 10 K. Furthermore, Supplementary equation 16 is derived for the 3D isotropic limit and may not be applicable to our strongly anisotropic crystal geometry. There are also likely to be errors in converting the conductances which we measure at low temperature into physically relevant conductivities. These originate from the possible presence of fluctuating/short-range charge order and the difficulty of estimating the effective cross-sectional areas of our crystals, given that the combination of disorder and extreme 1D anisotropy may restrict current flow to a fraction of the total crystal cross-section at low temperature.

Instead, it would be preferable if we could determine ξ_C from our VRH fits, since the parameters which we extract from our fitting provide an estimate of the disorder which should be robust to the problems and errors discussed above. From scaling theory, we know that the correlation length should obey the following relation on both the metallic and the insulating side of the transition:

$$\xi_C \propto |t - t_c|^{-\nu} \tag{17}$$

Here t is the control parameter allowing us to approach the Anderson transition (for example the disorder or the Fermi energy), t_c is the critical value of the parameter at which the transition occurs and ν is the critical exponent (which should not be confused with the VRH exponent $\nu = (1+d)^{-1}$). For the 3D orthogonal universality class [56], $\nu = 1.57$.

On the metallic side of the Anderson transition, our VRH T_0 provides a measure of the disorder (and is approximately proportional [57] to the inverse scattering time $1/\tau$). We therefore parametrise our disorder using T_0 as our control parameter and hence obtain:

$$\xi_C = c \left| T_0 - T_0^{\mathrm{crit}} \right|^{-\nu} \tag{18}$$

where c is a constant. Using $\left| T_0 - T_0^{\mathrm{crit}} \right|^{-\nu}$ as an effective correlation length, we may now fit T_{pk} using a rescaled version of Supplementary equation 15:

$$T_{\mathrm{pk}} = a\left[\left|T_0 - T_0^{\mathrm{crit}}\right|^{-1.57}\right]^{-3}\exp\left(-b\left[\left|T_0 - T_0^{\mathrm{crit}}\right|^{-1.57}\right]^{\Delta_2}\right) \tag{19}$$

where $a{\equiv}A/c^3$ and $b{\equiv}Bc^{\Delta_2}$ are constants. We must now determine the critical T_0 at the mobility edge, T_0^{crit}. Motivated by the fact that the localisation length is proportional to $T_0^{-1/d}$ within VRH theory, we plot T_{pk} vs. $T_0^{1/3}$ in Supplementary Fig. 8a. It is plausible that T_0^{crit} lies at the intersection of the two dashed lines linearly extrapolating the evolution of T_{pk} on each side of the transition, yielding $T_0^{\mathrm{crit}} \sim 223$ K. Alternatively (and more rigorously), we can define a wider range for T_0^{crit} – up to ~ 1500 K – using the "jump" in T_{pk} and T_0 at the mobility edge (Supplementary Fig. 8b).

In Supplementary Fig. 8c, we fit our data using Supplementary equation 19 for three values of T_0^{crit}: 223 K, 500 K and 1500 K, which roughly correspond to the two extremities of our estimated T_0^{crit} range from Supplementary Fig. 8b and their logarithmic average. For $T_0^{\mathrm{crit}} = 223$ K, the mobility edge lies close to crystal C: least-squares fitting with a, b and Δ_2 as free parameters yields $\Delta_2 = -0.075$. If we attempt to manually assign larger or smaller values to Δ_2, the fit fails (as demonstrated for $\Delta_2 = -0.2, -0.01$).

For $T_0^{\mathrm{crit}} = 500$ K, the metallic crystals lie further from the mobility edge and unconstrained fitting yields $\Delta_2 = -0.45$. Any attempt to fit our data using a fixed $\Delta_2 = -1.7$ (corresponding to the expected multifractal exponent in the isotropic 3D limit [58]) fails. In contrast, fitting with a small fixed $\Delta_2 = -0.01$ provides results which appear visually acceptable, with $b = 381$. However, we may rule out such small values of Δ_2 by considering the physical significance of $b = 381$. We recall that $b = Bc^{\Delta_2}$, where $B = c_3/|\gamma_{\mathrm{c},0}|$. Although $c_3 \sim \mathcal{O}(1)$, the bare Cooper interaction $|\gamma_{\mathrm{c},0}| \lesssim 1$. c is the proportionality constant linking $\left|T_0 - T_0^{\mathrm{crit}}\right|^{-1.57}$ to the true correlation length, which we expect to be of the order of 100 nm (by symmetry with crystals D,E,F on the insulating side of the q1D mobility edge): since $\left|T_0 - T_0^{\mathrm{crit}}\right|^{-1.57}$ is roughly 10^{-4} for $T_0^{\mathrm{crit}} = 500$, we estimate $c \sim 10^{-3}$. To check whether our fitted $\Delta_2 = -0.01$ and $b = 381$ are physically viable parameters, we evaluate $b = Bc^{\Delta_2}$ using a reasonable $|\gamma_{\mathrm{c},0}| = 0.1$ and obtain $b_{\mathrm{eval}} \sim 11$. This is much lower than $b_{\mathrm{fit}} = 381$ and hence $\Delta_2 = -0.01$ is inconsistent with our experimental data. However, if we evaluate b for the $\Delta_2 = -0.45$ fit, we obtain a similarly inconsistent $b_{\mathrm{eval}} = 224$ compared with $b_{\mathrm{fit}} = 0.1$. Therefore, the true Δ_2 clearly lies between -0.45 and -0.01. To confirm this statement, we fix $\Delta_2 = -0.1$ and obtain a much improved correspondence between $b_{\mathrm{eval}} = 20$ and $b_{\mathrm{fit}} = 24$.

Similar arguments apply to our fits with $T_0^{\mathrm{crit}} = 1500$ K, where we are sufficiently far from the mobility edge that $d^2T_{\mathrm{pk}}/d\xi_{\mathrm{C}}^2 > 0$ and our data can be reproduced by arbitrary values of Δ_2.

Evaluating b for a fixed trial $\Delta_2 = -0.45$, we obtain inconsistent values $b_{\rm eval} = 79$ versus $b_{\rm fit} = 0.1$. Again, this may be remedied by fixing $\Delta_2 = -0.1$, yielding mutually compatible $b_{\rm eval} = 16$ and $b_{\rm fit} = 24$.

The above analysis illustrates that although we know neither the absolute values of the correlation length on the metallic side of the localisation transition, nor the precise position of the mobility edge, we may nevertheless interpret our results within a multifractal enhancement scenario. The analytical formula derived in ref. [53] (Supplementary equation 15) provides a good description of our experimental data on the metallic side of the transition. However, the magnitude of the multifractal exponents which we obtain is substantially reduced relative to the 3D isotropic limit [58]: we estimate $\Delta_2 \sim -0.1$. This reduction may be attributed to the q1D symmetry of our crystals, which weakens the multifractality. For comparison, $\Delta_2 = -0.344$ for the symplectic-class Anderson transition in 2D. We therefore anticipate $|\Delta_2| < 0.344$ for a q1D material, in agreement with our data.

Recently, it has been shown that close proximity of a disordered superconductor to a medium with high dielectric constant is sufficient to screen the long-range Coulomb interaction, thus leading to the enhancement of superconductivity [51]. Although these calculations were carried out for 2D symmetry, similar arguments are likely to be valid in the highly anisotropic 3D limit relevant to $Na_{2-\delta}Mo_6Se_6$. While this enhancement still originates from multifractality in the local density of states, it is quantitatively unrelated to the properties of the non-interacting Anderson transition. This may also lead to the difference between our experimentally determined $\Delta_2 \sim$ -0.1 and the calculated $\Delta_2 =$ -1.7 for 3D Anderson localisation.

Supplementary Note IX. Distinguishing disorder-enhanced superconductivity from other mechanisms for raising T_c

Enhanced transition temperatures have been reported in many low-T_c superconductors since the 1960s and successfully attributed to a range of factors unrelated to disorder. Here, we briefly outline why none of these existing enhancement mechanisms for superconductivity appears to be compatible with our results.

T_c is known to rise by a factor of at least 3 for inhomogeneous aluminium films [59]. This was initially attributed to a surface-induced reduction in the phonon frequencies [60]; however this interpretation has since been cast into doubt, with localised magnetic moments at the grain boundaries [61] and clustering effects [62] possibly also contributing. In our view, nanoclustering must play some role in enhancing T_c for Al films, given that oscillations in T_c are seen as a function of film thickness [63]. Recently, an accurate description for the evolution of T_c in metallic nanoparticles has indeed been achieved using a combination of quantum confinement (i.e. finite size effects creating shape resonance peaks in the density of states) and mass renormalisation due to electron-phonon scattering [64].

Similar T_c enhancement mechanisms cannot apply to $Na_{2-\delta}Mo_6Se_6$: firstly, there is no evidence for Kondo-like spin scattering (e.g. logarithmic divergence in $\rho(T)$ at low temperature or negative $\rho(T)$ curvature at high temperature). Secondly, since we always measure single crystals (rather than granular or clustered films), any surface-induced phonon renormalisation effects on T_c should be identical for all our samples. Below the crystal surface, the presence of Na vacancies should not affect the MoSe intra-chain phonons which are responsible for superconductivity. In $Tl_2Mo_6Se_6$, an additional coupling to a Tl^+ optical phonon has been shown to increase T_c relative to $In_2Mo_6Se_6$. However, Na^+ is much smaller and lighter than either Tl^+ or In^+: since the electron-phonon coupling $\lambda_{e-ph} \propto 1/\omega^2$ and the phonon frequency ω scales as the square root of the M ion mass, the influence of the Na^+ phonon on T_c is expected to be negligible. We also note that there is no evidence in the granular/nanoparticle literature for any large enhancement in the pairing energy, and hence no precedent for the factor 4 increase in the Pauli limit in $Na_{2-\delta}Mo_6Se_6$ (Fig. 5e,g in the main text).

Impurities can increase T_J (at which Josephson coupling between 1D SC filaments occurs) [65], but this only helps to stabilise transverse phase coherence and has no effect on the pairing temperature T_{pk}. The transverse electron-phonon coupling is also known to rise in the presence of disorder [66]. However, this cannot influence the onset of 1D superconducting fluctuations at T_{pk}

in a q1D material where the wavevectors of the phonons responsible for superconductivity lie parallel to the 1D axis. Anderson U impurities have been proposed to locally increase electron-phonon coupling [67], but a Na vacancy cannot be considered a U impurity [68] since it merely leaves a small charge deficit to be screened on the MoSe chains, without any local U enhancement.

Although bulk Bi is a semi-metal at ambient pressure, it has been known since the 1950s that superconductivity with T_c exceeding 6 K may be induced in amorphous Bi films [69]. This is due to an enhancement in the density of states $N(E_F)$ within amorphous Bi compared to its crystalline form. No such enhancement of $N(E_F)$ occurs in our crystals: $Na_{2-\delta}Mo_6Se_6$ always remains crystalline and $N(E_F)$ is constant for Na deficiencies up to 25%, far greater than those achieved in our samples. Furthermore, increasing the disorder in amorphous Bi (by reducing the film thickness) monotonically suppresses T_c [70], in direct contrast to the behaviour of $Na_{2-\delta}Mo_6Se_6$.

$M_2Mo_6Se_6$ crystals are distant relatives of the 3D Chevrel phases MMo_6X_8 (X = S,Se) [71], which are composed of discrete Mo_6X_8 clusters rather than $(Mo_6Se_6)_\infty$ chains. T_c rises up to 15 K and H_{c2} exceeds 80 T in $PbMo_6S_8$; however, these properties are unrelated to the disorder-enhanced T_{pk} and H_{c2} which we report in the present work. In the Chevrel phases, the elevated values for T_c and H_{c2} are due to high densities of states and multiple bands crossing the Fermi level respectively [27]. The electronic structure of $M_2Mo_6Se_6$ is markedly different: $N(E_F)$ is more than an order of magnitude lower and a single 1D helix band crosses E_F. The properties of disordered $M_2Mo_6Se_6$ therefore cannot be quantitatively compared with the Chevrel phases.

In conclusion, the rise in T_{pk} which we observe in $Na_{2-\delta}Mo_6Se_6$ is inconsistent with any previously-observed enhancement mechanism for superconductivity. Instead, our results indicate a positive correlation between disorder-induced localisation and superconductivity.

SUPPLEMENTARY REFERENCES

[1] Petrović, A. P. *et al.* Phonon mode spectroscopy, electron-phonon coupling, and the metal-insulator transition in quasi-one-dimensional $M_2Mo_6Se_6$. *Phys. Rev. B* **82**, 235128 (2010).

[2] Ansermet, D. *et al.* Reentrant phase coherence in superconducting nanowire composites. *ACS Nano* **10**, 515–523 (2016).

[3] Chudzinski, P., Jarlborg, T. & Giamarchi, T. Luttinger-liquid theory of purple bronze $Li_{0.9}Mo_6O_{17}$ in the charge regime. *Phys. Rev. B* **86**, 075147 (2012).

[4] Giamarchi, T. *Quantum physics in one dimension* (Clarendon Press, Oxford, 2003).

[5] Armici, J. C. *et al.* A new pseudo-one-dimensional superconductor: $Tl_2Mo_6Se_6$. *Solid State Commun.* **33**, 607–611 (1980).

[6] Brusetti, R., Monceau, P., Potel, M., Gougeon, P. & Sergent, M. The exotic superconductor $Tl_2Mo_6Se_6$ investigated by low field magnetization measurements. *Solid State Commun.* **66**, 181–187 (1988).

[7] Brusetti, R., Briggs, A. & Laborde, O. Superconducting and dielectric instabilities in $Tl_2Mo_6Se_6$: Unusual transport properties and unsaturating critical field. *Phys. Rev. B* **49**, 8931–8943 (1994).

[8] Tarascon, J. M., DiSalvo, F. J. & Waszczak, J. V. Physical properties of several $M_2Mo_6X_6$ compounds. *Solid State Commun.* **52**, 227–231 (1984).

[9] Hor, P. H. *et al.* Study of the metal-semiconductor transition in $Rb_2Mo_6Se_6$, $Rb_2Mo_6Te_6$ and $Cs_2Mo_6Te_6$ under pressures. *Solid State Commun.* **55**, 231–235 (1985).

[10] Henrich, B. *et al.* PILATUS: A single photon counting pixel detector for X-ray applications. *Nuclear Instr. and Methods* **607**, 247-249 (2009).

[11] Dyadkin, V. SNBL Tool Box, Release 2012-1. Swiss Norwegian Beam Lines at ESRF, Grenoble, France (2012).

[12] Agilent Technologies, version 171.36.24.

[13] Sheldrick, G.M. SHELXL97. University of Gottingen, Germany (1997).

[14] Potel, M. *et al.* New pseudo-one-dimensional metals : $M_2Mo_6Se_6$ (M = Na, In, K, Tl), $M_2Mo_6S_6$ (M = K, Rb, Cs), $M_2Mo_6Te_6$ (M = In, Tl). *J. Solid State Chem.* **35**, 286–290 (1980).

[15] Tarascon, J. M., Hull, G. W. & DiSalvo, F. J. A facile synthesis of pseudo one-monodimensional ternary molybdenum chalcogenides $M_2Mo_6X_6$ (X=Se,Te; M=Li,Na..Cs). *Mater. Res. Bull.* **19**, 915–924 (1984).

[16] Hor, P. H., Meng, R. L., Chu, C. W., Tarascon, J. M. & Wu, M. K. High pressure study on quasi-one-dimensional compounds $M_2Mo_6X_6$. *Physica* **135B**, 245–247 (1985).

[17] Imada, M., Fujimori, A. & Tokura, Y. Metal-insulator transitions. *Rev. Mod. Phys.* **70**, 1039–1263 (1998).

[18] Mott, N. F. Conduction in non-crystalline materials. *Phil. Mag.* **19**, 835–852 (1969).

[19] Efros, A. L. & Shklovskii, B. I. Coulomb gap and low temperature conductivity of disordered systems. *J. Phys. C Solid State Phys.* **8**, L49–L51 (1975).

[20] Martin, I. & Phillips, P. Positive magnetoresistance in quasi-one-dimensional conductors. *Phys. Rev. B* **56**, 14883–14885 (1997).

[21] Fukuyama, H. & Yosida, K. Negative magnetoresistance in the Anderson localized states. *J. Phys. Soc. Jpn.* **46**, 102–105 (1979).

[22] Gershenson, M. E., Khavin, Y. B., Mikhalchuk, A. G., Bozler, H. M. & Bogdanov, A. L. Crossover from weak to strong localization in quasi-one-dimensional conductors. *Phys. Rev. Lett.* **79**, 725–728 (1997).

[23] Sharifi, F., Herzog, A. V. & Dynes, R. C. Crossover from two to one dimension in *in situ* grown wires of Pb. *Phys. Rev. Lett.* **71**, 428–431 (1993).

[24] Larkin, A. I. & Varlamov, A. A. Fluctuation phenomena in superconductors. In Bennemann, K. & Ketterson, J. (eds.) *Superconductivity*, 369–458 (Springer Berlin Heidelberg, 2008).

[25] Fogler, M. M., Teber, S. & Shklovskii, B. I. Variable-range hopping in quasi-one-dimensional electron crystals. *Phys. Rev. B* **69**, 035413 (2004).

[26] Aoki, H. Fractal dimensionality of wave functions at the mobility edge: Quantum fractal in the Landau levels. *Phys. Rev. B* **33**, 7310–7313 (1986).

[27] Petrović, A. P. *et al.* Multiband superconductivity in the Chevrel phases $SnMo_6S_8$ and $PbMo_6S_8$. *Phys. Rev. Lett.* **106**, 017003 (2011).

[28] Inada, R., Onuki, Y. & Tanuma, S. Paramagnetic property in the commensurate charge density wave phase of 1T-TaS_2. *J. Phys. Soc. Jpn.* **50**, 1217–1221 (1981).

[29] Langer, J. S. & Ambegaokar, V. Intrinsic resistive transition in narrow superconducting channels. *Phys. Rev.* **164**, 498–510 (1967).

[30] McCumber, D. E. & Halperin, B. I. Time scale of intrinsic resistive fluctuations in thin superconducting wires. *Phys. Rev. B* **1**, 149–157 (1970).

[31] Lau, C. N., Markovic, N., Bockrath, M., Bezryadin, A. & Tinkham, M. Quantum phase slips in superconducting nanowires. *Phys. Rev. Lett.* **87**, 217003 (2001).

[32] Cirillo, C. *et al.* Quantum phase slips in superconducting Nb nanowire networks deposited on self-assembled Si templates. *Appl. Phys. Lett.* **101**, 172601 (2012).

[33] Arutyunov, K. Y., Golubev, D. S. & Zaikin, A. D. Superconductivity in one dimension. *Phys. Rep.* **464**, 1–70 (2008).

[34] Golubev, D. S. & Zaikin, A. D. Quantum tunneling of the order parameter in superconducting nanowires. *Phys. Rev. B* **64**, 014504 (2001).

[35] Ghosal, A., Randeria, M. & Trivedi, N. Inhomogeneous pairing in highly disordered *s*-wave superconductors. *Phys. Rev. B* **65**, 014501 (2001).

[36] Dubi, Y., Meir, Y. & Avishai, Y. Nature of the superconductor-insulator transition in disordered superconductors. *Nature* **449**, 876–80 (2007).

[37] Feigel'man, M. V., Ioffe, L. B., Kravtsov, V. E. & Yuzbashyan, E. A. Eigenfunction fractality and pseudogap state near the superconductor-insulator transition. *Phys. Rev. Lett.* **98**, 027001 (2007).

[38] Bouadim, K., Loh, Y. L., Randeria, M. & Trivedi, N. Single- and two-particle energy gaps across the disorder-driven superconductor-insulator transition. *Nat. Phys.* **7**, 884–889 (2011).

[39] Petrović, A. *et al.* Unconventional resistive transitions in the extreme type-II superconductor $Tl_2Mo_6Se_6$. *Phys. C Supercond.* **460-462**, 702–703 (2007).

[40] Mishonov, T. M., Pachov, G. V., Genchev, I. N., Atanasova, L. A. & Damianov, D. C. Kinetics and Boltzmann kinetic equation for fluctuation Cooper pairs. *Phys. Rev. B* **68**, 054525 (2003).

[41] Kadin, A. M., Epstein, K. & Goldman, A. M. Renormalization and the Kosterlitz-Thouless transition in a two-dimensional superconductor. *Phys. Rev. B* **27**, 6691–6702 (1983).

[42] Bergk, B. *et al.* Superconducting transitions of intrinsic arrays of weakly coupled one-dimensional superconducting chains: the case of the extreme quasi-1D superconductor $Tl_2Mo_6Se_6$. *New J. Phys.* **13**, 103018 (2011).

[43] Wang, Z., Shi, W., Lortz, R. & Sheng, P. Superconductivity in 4-Angstrom carbon nanotubes - a short review. *Nanoscale* **4**, 21–41 (2012).

[44] Sun, M. Y. *et al.* Dimensional crossover transition in a system of weakly coupled superconducting nanowires. *New J. Phys.* **14**, 103018 (2012).

[45] He, M. *et al.* 1D to 3D dimensional crossover in the superconducting transition of the quasi-one-dimensional carbide superconductor Sc_3CoC_4. *J. Phys. Condens. Matter* **27**, 075702 (2015).

[46] Benfatto, L., Castellani, C. & Giamarchi, T. Broadening of the Berezinskii-Kosterlitz-Thouless superconducting transition by inhomogeneity and finite-size effects. *Phys. Rev. B* **80**, 214506 (2009).

[47] Feigel'man, M. V., Ioffe, L. B., Kravtsov, V. E. & Cuevas, E. Fractal superconductivity near localization threshold. *Ann. Phys. (N. Y).* **325**, 1390–1478 (2010).

[48] Tezuka, M. & García-García, A. M. Stability of the superfluid state in a disordered one-dimensional ultracold fermionic gas. *Phys. Rev. A* **82**, 043613 (2010).

[49] Kravtsov, V. E. Wonderful life at weak Coulomb interaction: increasing of superconducting/superfluid transition temperature by disorder. *J. Phys. Conf. Ser.* **376**, 012003 (2012).

[50] Mayoh, J. & García-García, A. M. Global critical temperature in disordered superconductors with weak multifractality. *Phys. Rev. B* **92**, 174526 (2015).

[51] Burmistrov, I. S., Gornyi, I. V. & Mirlin, A. D. Superconductor-insulator transitions: Phase diagram and magnetoresistance. *Phys. Rev. B* **92**, 014506 (2015).

[52] Clogston, A. M. Upper limit for the critical field in hard superconductors. *Phys. Rev. Lett.* **9**, 266–267 (1962).

[53] Burmistrov, I. S., Gornyi, I. V. & Mirlin, A. D. Enhancement of the critical temperature of superconductors by Anderson localization. *Phys. Rev. Lett.* **108**, 017002 (2012).

[54] Aoki, H. Critical behaviour of extended states in disordered systems. *J. Phys. C Solid State Phys.* **16**, L205–L208 (1983).

[55] Schreiber, M. & Grussbach, H. Multifractal wave functions at the Anderson transition. *Phys. Rev. Lett.* **67**, 607–610 (1991).

[56] Slevin, K. & Ohtsuki, T. Critical exponent for the Anderson transition in the three-dimensional orthogonal universality class. *New J. Phys.* **16**, 015012 (2014).

[57] Knyazev, D., Omelyanovskii, O., Pudalov, V. & Burmistrov, I. Metal-insulator transition in two dimensions: experimental test of the two-parameter scaling. *Phys. Rev. Lett.* **100**, 046405 (2008).

[58] Evers, F. & Mirlin, A. D. Anderson transitions. *Rev. Mod. Phys.* **80**, 1355–1417 (2008).

[59] Abeles, B., Cohen, R. W. & Cullen, G. W. Enhancement of superconductivity in metal films. *Phys. Rev. Lett.* **17**, 632–634 (1966).

[60] Hauser, J. Enhancement of superconductivity in aluminium films. *Phys. Rev. B* **3**, 1611–1616 (1971).

[61] Bachar, N., Lerer, S., Hacohen-Gourgy, S., Almog, B. & Deutscher, G. Kondo-like behavior near the metal-to-insulator transition of nanoscale granular aluminum. *Phys. Rev. B* **87**, 214512 (2013).

[62] Cao, B. *et al.* Evidence for high T_c superconducting transitions in isolated Al_{45} and Al_{47} nanoclusters. *J. Supercond. Nov. Magn.* **21**, 163–166 (2008).

[63] Liu, Y., Haviland, D., Nease, B. & Goldman, A. Insulator-to-superconductor transition in ultrathin films. *Phys. Rev. B* **47**, 5931–5946 (1993).

[64] Croitoru, M. D. *et al.* Phonon limited superconducting correlations in metallic nanograins. *Sci. Rep.* **5**, 16515 (2015).

[65] Efetov, K. B. & Larkin, A. I. Effect of fluctuations on the transition temperature in quasi-one-dimensional superconductors. *Sov. Phys. JETP* **39**, 1129–1134 (1974).

[66] Keck, B. & Schmid, A. Superconductivity and electron-phonon interaction in impure simple metals. *J. Low Temp. Phys.* **24**, 611–629 (1976).

[67] Martin, I. & Phillips, P. Local pairing at U impurities in BCS superconductors can enhance T_c. *Phys. Rev. B* **56**, 14650–14654 (1997).

[68] Anderson, P. W. Localized magnetic states in metals. *Phys. Rev.* **124**, 41–53 (1961).

[69] Buckel, W. Elektronenbeugungs-Aufnahmen von dünnen Metallschichten bei tiefen Temperaturen. *Zeitschrift für Phys.* **138**, 136–150 (1954).

[70] Haviland, D. B., Liu, Y. & Goldman, A. M. Onset of superconductivity in the two-dimensional limit. *Phys. Rev. Lett.* **62**, 2180–2183 (1989).

[71] Fischer, Ø. Chevrel phases: superconducting and normal state properties. *Appl. Phys.* **16**, 1–28 (1978).
